# Genome-Wide Identification and Expression Profiling of the Polygalacturonase (*PG*) and Pectin Methylesterase (*PME*) Genes in Grapevine (*Vitis* *vinifera* L.)

**DOI:** 10.3390/ijms20133180

**Published:** 2019-06-28

**Authors:** Nadeem Khan, Fizza Fatima, Muhammad Salman Haider, Hamna Shazadee, Zhongjie Liu, Ting Zheng, Jinggui Fang

**Affiliations:** 1 State Key Laboratory of Crop Genetics and Germplasm Enhancement, Ministry of Science and Technology/College of Horticulture, Nanjing Agricultural University, Nanjing 210095, China; 2 Ottawa Research and Development Centre, Agriculture and Agri-Food Canada, Ottawa, ON K1A 0C6, Canada; 3 Key laboratory of Genetics and Fruit Development, College of Horticulture, Nanjing Agricultural University, Nanjing 210095, China; 4 College of Life Science, Nanjing Agricultural University, Nanjing 210095, China

**Keywords:** polygalacturonase (PGs), pectin methylesterase (PMEs), collinearity analysis, gene duplications, expression profiling, grapevine

## Abstract

In pectin regulation, polygalacturonases (PGs) and pectin methylesterases (PMEs) are critical components in the transformation, disassembly network, and remodeling of plant primary cell walls. In the current study, we identified 36 *PG* and 47 *PME* genes using the available genomic resources of grapevine. Herein, we provide a comprehensive overview of PGs and PMEs, including phylogenetic and collinearity relationships, motif and gene structure compositions, gene duplications, principal component analysis, and expression profiling during developmental stages. Phylogenetic analysis of PGs and PMEs revealed similar domain composition patterns with *Arabidopsis*. The collinearity analysis showed high conservation and gene duplications with purifying selection. The type of duplications also varied in terms of gene numbers in PGs (10 dispersed, 1 proximal, 12 tandem, and 13 segmental, respectively) and PMEs (23 dispersed, 1 proximal, 16 tandem, and 7 segmental, respectively). The tissue-specific response of *PG* and *PME* genes based on the reported transcriptomic data exhibited diverged expression patterns in various organs during different developmental stages. Among PGs, *VvPG8, VvPG10, VvPG13, VvPG17, VvPG18, VvPG19, VvPG20, VvPG22*, and *VvPG23* showed tissue- or organ-specific expression in majority of the tissues during development. Similarly, in PMEs, *VvPME3, VvPME4, VvPME5, VvPME6, VvPME19, VvPME21, VvPME23, VvPME29, VvPME31*, and *VvPME32* suggested high tissue-specific response. The gene ontology (GO), Kyoto Encyclopedia of Genes and Genomics (KEGG) enrichment, and cis-elements prediction analysis also suggested the putative functions of PGs and PMEs in plant development, such as pectin and carbohydrate metabolism, and stress activities. Moreover, qRT-PCR validation of 32 *PG* and *PME* genes revealed their role in various organs of grapevines (i.e., root, stem, tendril, inflorescence, flesh, skins, and leaves). Therefore, these findings will lead to novel insights and encourage cutting-edge research on functional characterization of PGs and PMEs in fruit crop species.

## 1. Introduction

Plant cell walls are mainly composed of various interacting networks of carbohydrate polymers, such as polymers of cellulose, hemicellulose, and pectins [1]. These are produced by the plant cells before being systemically delivered to the apoplast tissues [2], providing support and mechanical strength to the plants. However, the disruption of the cell wall structure exposes fruit crops to disorders, such as softening and susceptibility to diseases [3]. Until now, several studies have shown multiple associated roles linked to pectin degradation enzymes in plants. In particular, polygalacturonase (PGs; EC 3.2.1.15) hydrolytic enzymes are involved in plant organ cell separation events, reproductive developments, leaf morphology, and organ shedding [4,5,6,7]. Furthermore, expression profiling, both at a temporal and spatial level, has revealed that transcript accumulations are also responsible for cell wall softening during abscission, ripening, and dehiscence [8]. Moreover, these correlation analyses are useful for identifying cell wall sites during disassembly and studying the transcriptional patterns of key genes in cell-wall degrading enzymes [5]. In *Arabidopsis,* during different developmental stages, several members of PGs can be detected by real-time PCR (RT-PCR) in various organs, such as roots, leaves, pollen tubes, flowers, and siliques [9,10]. In addition, few members of PGs have been functionally characterized in some fruit crop species, such as grapevine (*VvPG1* and *VvPG2*), apple (*MdPG36*), banana (*MAPG1* to *MAPG4*), and pear (*PcPGl* and *PcPG3*) [11,12,13,14]. However, the silencing of *FaPG1* gene in strawberries declines the halt of the middle lamella and reduces fruit softening [15]. In pears and bananas, the softening of fruits is regulated by *Pc-PG1* and *Pc-PG2* as well as *MaPG3* and *MaPG4*, in an ethylene-dependent pattern [16,17]. 

Pectin methylesterases (PMEs; EC 3.1.1.11) are also hydrolytic enzymes present in plants, which play a pivotal role in the firmness and softening of the cell walls, specifically through remodeling and disassembly of pectin [18,19]. The PMEs also play a crucial role in plant development, including fruit maturity, pollen development, pollen tube growth, and other growth-related factors [20,21]. However, the activity of both PGs and PMEs during fruit softening is largely unknown and various studies have shown their contradictory actions [22,23]. PGs in plants contain three groups, namely class A, B, and C, whereas the PMEs are categorized into two main types, namely type I and type II [19,24]. PGs and PMEs are a major gene family and have extensively been studied in various crops, including *Brassica rapa, Gossypium* species, *Arabidopsis thaliana*, *Solanum lycopersicum*, *Malus domestica*, *Cucumis sativus*, and *Citrullus lanatus* [5,11,25,26,27,28]. 

Grapevine (*Vitis vinifera* L.) is one of the most important woody fruit crop resource in the world [29]. They have become widely popular due to their nutritional value and health benefits. Improving the grapevine berry quality is a crucial issue, and the availability of the grapevine genome (Version 2.1) provides an excellent opportunity for its genetic study, momentously facilitating research in grapevine biology. In grapevine, the members of PGs and PMEs have not yet been comprehensively identified by genome-wide analysis and their elucidation is largely unknown. In this study, we utilized genomic resources to characterize PGs and PMEs in grapevine by various bioinformatic tools. In total, 36 *PG* and 47 *PME* genes were identified in grapevine and compared with *Arabidopsis* in order to test their domain composition. The expression patterns of genes provide valuable clues for understanding their functions. We also tested 32 *PG* and *PME* genes for various organs of grapevine, and their correlation factors were further compared. Overall, our study provides a brief understanding of the pectin-related genes in grapevine, and their substantial role in regulating organs during different developmental stages.

## 2. Results

### 2.1. Identification of PGs and PMEs in Grapevine

A total of 36 *PG* and 47 *PME* genes were identified from the grapevine genome through orthologues analysis with *Arabidopsis* (Table S1). These genes were designated as *VvPG1-VcPG36* for PGs and *VvPME1-VcPME46* for PMEs according to the previously reported nomenclature system with slight modification. The corresponding UNIPROT gene IDs are also listed in Table 1. A brief summary of the basic information for both PGs and PMEs in grapevine, apple, peach, and citrus is shown in Tables S1 and S2, where the basic features include the protein identifier, CDS length (bp), and protein properties (i.e., protein length (aa), molecular weight (kDa), isoelectric point (PIs), and grand average of hydropathicity (GRAVY)). Additionally, gene duplication types and subcellular predictions were also explored for each protein of PGs and PMEs in grapevine. In general, the coding sequence length of these genes varied from 999–3702 bp for PGs and 768–2463 bp for PMEs, while the protein length ranged from 332–1233 aa for PGs and 255–820 aa for PMEs, respectively. Moreover, the kDa ranged from 36.38–135.75 for PGs and 28.12–90.85 for PMEs, while the PIs varied from 4.73–9.74 for PGs and 5.17–9.97 for PMEs. Additionally, the results of GRAVY reveal that both PGs and PMEs are hydrophilic and hydrophobic in nature. Although most showed hydrophobic properties with negative values, a limited number showed positive hydrophobic properties. Protein subcellular predications also confirmed that the majority of PGs and PMEs occurred in the nucleus, endoplasmic reticulum, cytoplasm, plasma membrane, and mitochondria among ohers, which are listed in Table S1. Moreover, the types of duplications also varied in terms of gene numbers in PGs (10 dispersed, 1 proximal, 12 tandem, and 13 segmental, respectively) and PMEs (23 dispersed, 1 proximal, 16 tandem, and 7 segmental, respectively). Hence, the observed variations in various properties among PGs and PMEs implies that these genes may function contrarily in a variable environment. 

### 2.2. Phylogenetic and Collinearity Relationships, Motif Compositions and Genomic Structure of PGs and PMEs in Grapevine

**Figure 1:**
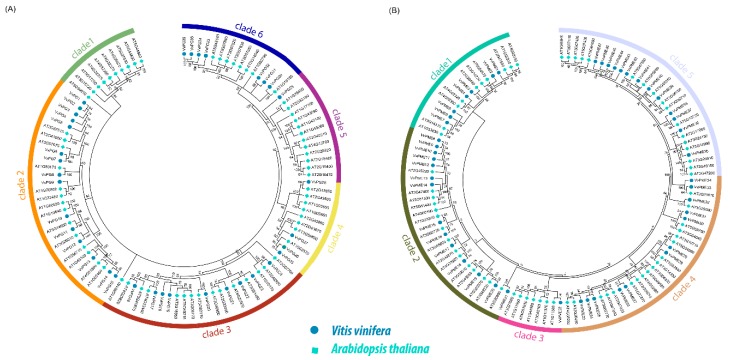
Phylogenetic relationship of *PG* (**A**) and *PME* (**B**) genes between grapevine and *Arabidopsis*. The phylogenetic tree was constructed by MEGA 7.0 using the Maximum Likelihood Method (1000 bootstrap).

**Figure 2:**
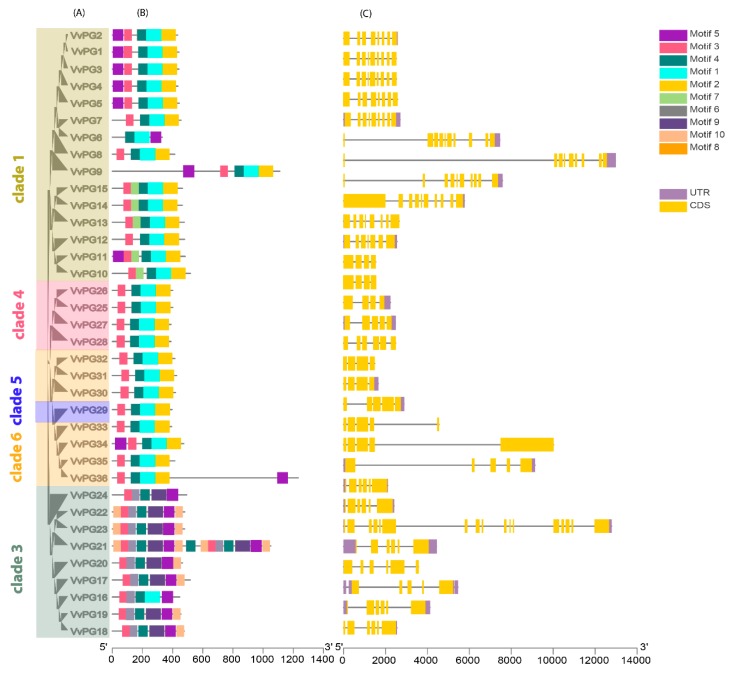
Phylogenetic relationship of *PGs* (**A**). The phylogenetic tree was constructed by MEGA 7.0 using the Maximum Likelihood Method (1000 bootstrap). Motif structure and upstream/downstream regions of *PGs* (**B**). The coding sequences (CDS) and untranslated regions (UTR) for PGs in grapevine (**C**). CDS and UTR are represented by yellow and green boxes. The relative position is proportionally displayed based on the kilobase scale at the bottom of the figures.

**Figure 3:**
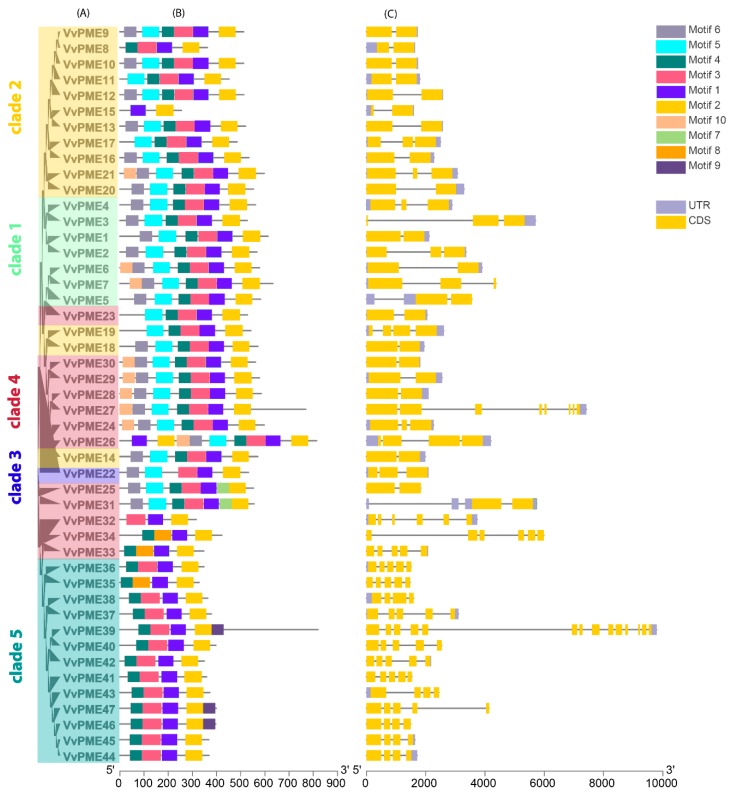
Phylogenetic relationship of *PMEs* (**A**). The phylogenetic tree was constructed by MEGA 7.0 using the Maximum Likelihood Method (1000 bootstrap). Motif structure and upstream/downstream regions of *PMEs* (**B**). The coding sequences (CDS) and untranslated regions (UTR) for PMEs in grapevine (**C**). CDS and UTR are represented by yellow and green boxes, respectively. The relative position is proportionally displayed based on the kilobase scale at the bottom of the figures.

The phylogenetic relationships of 36 PGs and 47 PMEs of grapevine and *Arabidopsis* were obtained using MEGA 7.0 with a maximum likelihood approach (ML). The phylogenetic tree revealed that *PG* and *PME* genes can further be divided into six and five major clades (Figure 1a,b). In the phylogenetic tree of both PGs and PMEs, we observed that clade two contained the most number of genes (15 and 14) compared to other clades in grapevine. The phylogenetic analysis suggests that both PGs and PMEs share high similarities and have close genetic relationships with *Arabidopsis*. The observed results of our phylogenetic arrangement were also consistent with previously reported studies [25,27]. In addition, we also constructed phylogenetic trees among *PG* and *PME* genes (Figure 2a and Figure 3a). The results showed consistency among the clades of PGs compared to PMEs, which may be due to variations in tree topologies. We also analyzed the composition of motifs for both PGs and PMEs (Figure 2b and Figure 3b). For PG and PME proteins, we obtained ten conserved motifs using the online server, Multiple Em for Motif Elicitation (MEME). The results revealed that motifs five and two frequently occurred among PG members. Similarly, for PME members, motifs six, five, four, and one were dominantly found in grapevine (Figure 2b and Figure 3b). Hence, these results suggest that most PG and PME protein members carry unique features due to variation in their amino acid sequences. Additionally, we also obtained their LOGOS by the same online server MEME. Ten consensus sequences were acquired for both PG and PME protein members and their distribution patterns are shown in Figures S1 and S2. 

Furthermore, based on coding sequence (CDS) and untranslated regions (UTR) of *PG* and *PME* genes in grapevine, gene structures were also resolved using TBtools software (Figure 2c and Figure 3c). The results revealed that both PG and PME members exhibited high divergence and were largely conserved compared to each other. On the other hand, the *PG* and *PME* genes displayed more or fewer similarities among the same clades. This was also observed in a previously reported study focusing on PGs and PMEs in *Brassica rapa* [25].

### 2.3. Chromosomal Localization and Gene Duplication Analysis of PG and PME Genes

**Figure 4:**
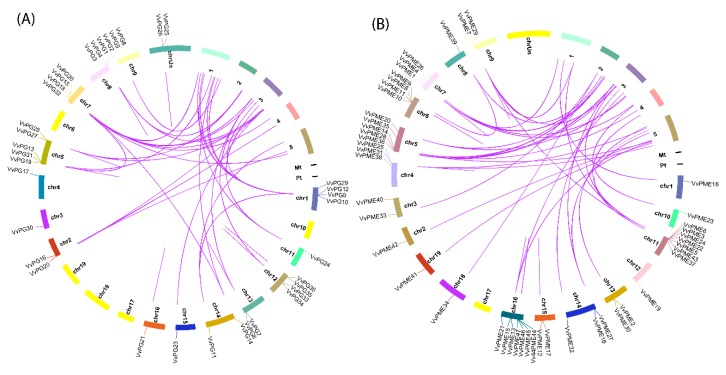
The collinear correlation for all genes of PGs (**A**) and PMEs (**B**) is displayed between grapevines and *Arabidopsis*. The localization of chromosomes was shown for grapevine and *Arabidopsis* in different random colors.

A total of 36 *PG* genes were distributed unevenly across different chromosomal locations of grapevine genomes (i.e., Chr01–Chr19). The majority of the chromosomes of PGs showed inconsistency in terms of genes. Both Chr05 and Chr08 exhibited the highest number (8) of genes, followed by Chr1 and Chr07 with five genes. The others varied in number (Figure 4a and Figure S3). Moreover, the chromosomal localization for PME members also displayed high variation in the number of genes. The highest number of genes (seven) was found on both Chr11 and Chr16 each, followed by Chr5 containing five genes. The others largely varied from 1–4 per chromosome (Figure 4b and Figure S3). In majority, we observed non-random distribution patterns of *PG* and *PME* genes in the grapevine genome. The 36 *PG* and 47 *PME* genes were also clustered for collinearity between grapevine and *Arabidopsis* using Circos (Figure 4a,b). The results illustrated high conservation among PME members compared to PGs.

To study evolutionary rates and types of duplications among *PG* and *PME* genes in grapevines, we used MEGA7.0 and MCScanX. Among the 36 PGs, we identified 10 dispersed, 1 proximal, 12 tandem, and 13 segmental genes. Furthermore, we determined 23 dispersed, 1 proximal, 16 tandem, and 7 segmental genes in the 47 PMEs (Table 1). As gene duplications are vital for discovering novel biological functions, evolutions, and gene expansion [30], the segmental and dispersed duplications observed may play a major role in the expansion of PG and PME members. 

To estimate the selection pressure among various types of duplications for both PGs and PMEs, we also intended their synonymous (*Ks*) and non-synonymous substitution rate (*Ka*) values. During evolutionary implications, genes are typically exposed to different types of selection processes (i.e., positive, neutral, and purifying selection). For understanding these selection pressures, we selected 15 duplicated pairs of genes among PGs and PMEs (Table 1). The *Ka/Ks* ratios for most PGs and PMEs were less than one, implicating a purifying selection and a reduction in divergence after duplications. However, we also found four pairs of PGs (i.e., *VvPG29-VvPG30*, *VvPG5-VvPG7*, *VvPG14-VvPG15*, and *VvPG18-VvPG21*) and five pairs of PMEs (*VvPME5-VvPME16*, *VvPME22-VvPME23*, *VvPME35-VvPME36*, *VvPME6-VvPME7*, and *VvPME14-VvPME26*) that indicate positive selection.

### 2.4. Gene Ontology Enrichment (GO) and Cis-Regulatory Elements in Grapevine

To study the regulatory functions of the 36 PGs and 47 PMEs, we performed GO annotation and GO enrichment analyses. The GO terms were largely based on three groupings, including molecular functions (MF), cellular component (CC), and biological process (BP). In brief, GO enrichments validate that PGs are enriched in various MF terms, such as “polygalacturonase activity” (GO:0004650), “hydrolase activity” (GO:0016787), and “hydrolyzing O-glycosyl compounds” (GO:0016798). The term CC was enriched in the plant-type cell wall, such as “integral component of membrane” (GO:0016021), and “extracellular region” (GO:0005576). The BP term was mainly responsive, such as “carbohydrate metabolic processes” (GO:0005975) and various other “metabolic processes” (GO:0008152), which are briefly listed in Table S3. Moreover, the PME results reveal that MF is enriched in “enzyme inhibitor activity” (GO:0004857) “pectinesterase activity” (GO:0030599), “aspartyl esterase” (GO:0045330). The CC and BP were also found to be enriched in “membrane” (GO:0016020), “proteolysis” (GO:0006508), and “cell wall modification” (GO:0042545). Our results from the GO enrichment analysis further hinted the role of PG and PME members in grapevine. 

The 36 *PG* and 47 *PME* genes were also tested for pathway enrichment analysis using the KEGG database, where the results showed enrichment in three major pathways (Table S4). These pathways include “carbohydrate metabolism” followed by “metabolism” and “pentose and glucuronate interconversions” in grapevines.

**Figure 5:**
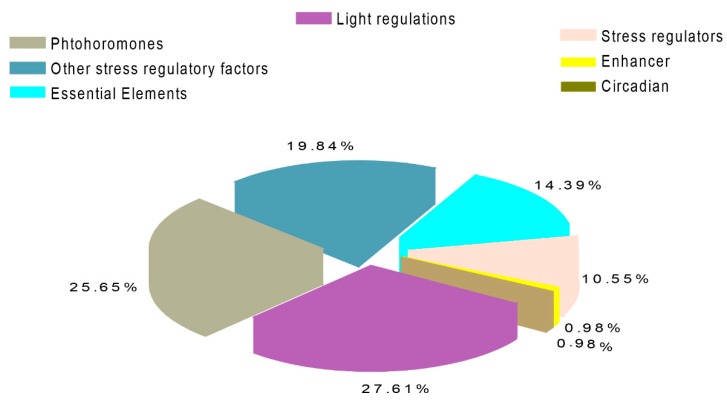
Various cis-elements identified in grapevine by using PlantCARE.

In addition, we also observed cis-acting elements by utilizing the promoter regions of both PG and PME members using the PlantCARE database. The various types of cis-regulatory elements were analyzed and are described in Figure 5 and Table S5. In brief, the majority of the genes participated in numerous signaling pathways, such as phytohormones, biotic-abiotic and other regulatory stress factors. For instance, 27.61% of the genes of PGs and PMEs were responsive to light regulations (e.g., GTI-motif, G-Box, GATA-motif, and others), followed by phytohormones (25.65%) (CGTCA, TGACG, ABRE). Other observed key regulatory elements include TC-Rich repeats, and HD-ZIP 3, which were reactive to defense stress and protein binding, respectively. These results inferred that the *PG* and *PME* genes have diverse gene functions and are indirectly involved in various biotic-abiotic/hormone signaling. 

### 2.5. Tanscriptional Profiling of PGs and PMEs in Different Organs and Developmental Stages in Grapevine 

**Figure 6:**
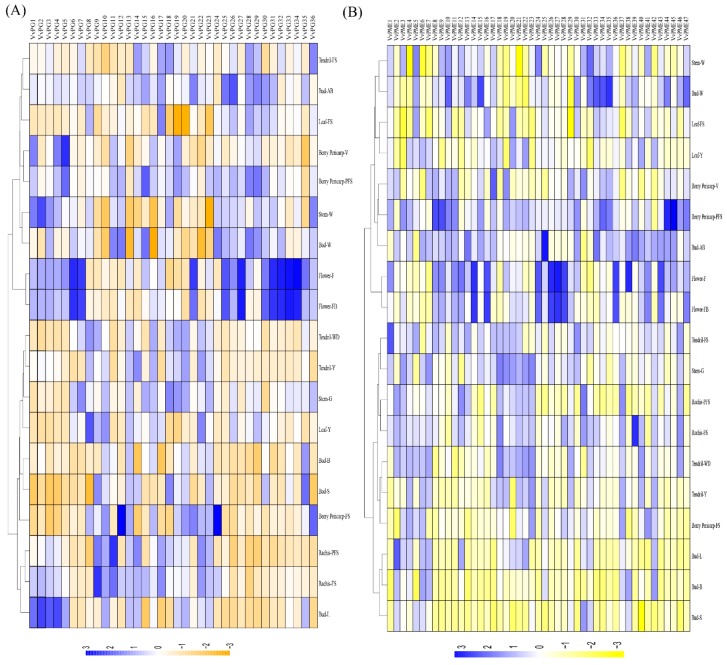
Expression profiles of the *PG* (**A**) and *PME* (**B**) genes in different grapevine organs, tissues, and, developmental stages. Data were normalized based on the mean expression values of each gene in all analyzed tissues. BerryPericarp-FS: berry pericarp fruit set; BerryPericarp-PFS: berry pericarp post-fruit set; BerryPericarp-V: Bud-S: bud swell; Bud-B: bud burst (green tip); Bud-AB: bud after-burst (rosette of leaf tips visible); Bud-L: latent bud; Bud-W: winter bud; Flower-FB: flowering begins (10% caps off); Flower-F: flowering (50% caps off); Leaf-Y: young leaf (pool of leaves from shoot of 5 leaves); Leaf-FS: mature leaf (pool of leaves from shoot at fruit set); Rachis-FS: rachis fruit set; Rachis-PFS: rachis post fruit set; Stem-G: green stem; Stem-W: woody stem; Tendril-Y: young tendril (pool of tendrils from shoot of 7 leaves); Tendril-WD: well developed tendril (pool of tendrils from shoot of 12 leaves); and Tendril-FS: mature tendril (pool of tendrils at fruit set).

To understand the spatiotemporal expression levels of *PG* and *PME* genes in grapevines, the global transcriptomic data of developmental phases of 19 different tissues and organs were retrieved from NCBI (GSE36128) [31]. Figure 6a,b represent the heat maps, indicating expression patterns of PGs and PMEs in grapevines. Among PGs, *VvPG8, VvPG10, VvPG13, VvPG17, VvPG18, VvPG19, VvPG20, VvPG22,* and *VvPG23* showed tissue- or organ-specific expression across many tissues during development. In contrast, the remaining PGs demonstrated weak tissue-specific response in any of the selected grapevine organs. Likewise, in PMEs, *VvPME3, VvPME4, VvPME5, VvPME6, VvPME19, VvPME21, VvPME23, VvPME29, VvPME31*, and *VvPME32* suggested higher tissue-specific response in all the tissues. In contrast, the others showed either moderate to weak expression or no expression in any of the grapevine tissues (Figure 6b). Overall the *PG* and *PME* genes showed enriched expression in flower (*VvPG6-7*, *VvPG31-34*, *VvPME-28*) and fruit ripening (*VvPG4-5*, *VvPME7-8*, and *VvPME44-45*), where the PGs suggested a more profound response than PMEs.

### 2.6. qRT-PCR Analysis of the Candidate PG and PME Genes in Various Organs of Grapevine

**Figure 7:**
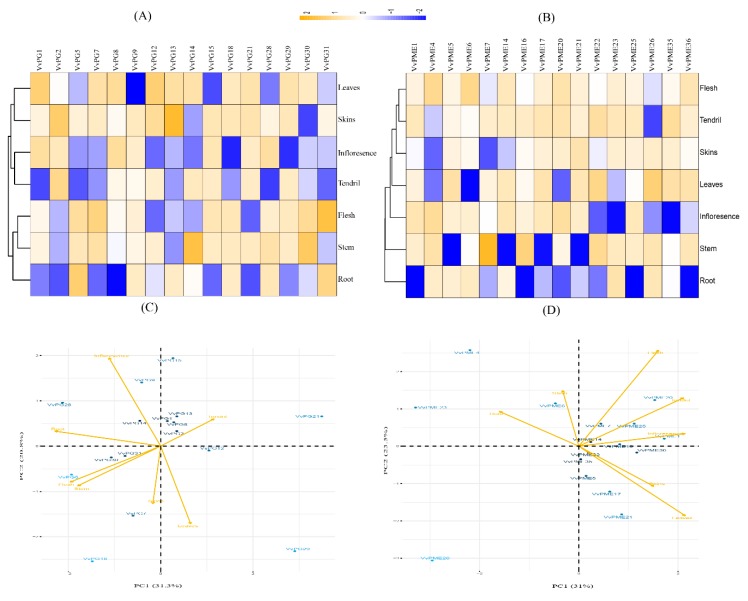
Relative expressions of PGs (**A**) and PMEs (**B**) in various organs, including root, stem, tendril, inflorescence, flesh, skins, and leaves and their principal component analysis for PGs (**C**) and PMEs (**D**).

To validate previous findings based on RNA-seq data, cis-element predications and gene duplication analysis, we randomly selected 32 genes for qRT-PCR, which showed either positive or purifying selection. The expression profiling of *PG* and *PME* genes was quantified in various organs, such as root, stem, tendril, inflorescence, berry flesh, berry skin, and leaf of grapevines (Figure 7a,b). The results suggest that PG and PME transcripts show distinct expression patterns, intimating that both (*PG* and *PME*) gene families have positive regulatory roles in various physiological processes in grapevine. Moreover, principal component analysis (PCA) analysis was performed to gain deeper insight into their contribution to organ development. PCA analysis of PG transcripts suggested a variation of 31.26% in PC1, 24.28% in PC2, and 15.36% in PC3, which accounted for 70.90% of the total variation in the first three axes (Figure 7c). Among PGs, *VvPG31* (0.86) and *VvPG5* (0.85) had high positive loadings, while *VvPG21* (−0.90) and *VvPG8* (−0.77) had high negative loadings in PC1. Moreover, PCA analysis of PME transcripts suggested 83.02% in first three axes (PC1, PC2, and PC3) (Figure 7d). Among the PME transcripts, *VvPME5* (0.73) and *VvPME14* (0.61) had high positive loadings in PC1, whereas the highest negative loadings were found in *VvPME16* (−0.96), *VvPME36* (−0.82) and *VvPME1* (−0.79) (Table S6).

## 3. Discussion

Realizing the significant role of PGs and PMEs in various plants observed in past studies, it is essential to systematically investigate the potential functions of these genes in grapevine. For instance, polygalacturonases (PGs) and pectin methylesterases (PMEs) have been hypothesized to play an imperative part in plant life cycles, such as cell separation and expansion, dehiscence, abscission, fruit maturity, and plant shedding [8,21,24]. In particular, PGs are a vital component of pectin disassembly, whereas PMEs play a central role in both remodeling and pectin disassembly [19,32]. Thus far, PGs and PMEs have been identified in various crops species although there is a lack of systemic analysis in grapevines. In this study, we comprehensively carried out various bioinformatics analyses by utilizing the available genomic resources of grapevines. In total, 36 *PG* and 47 *PME* genes were identified in grapevines and compared with *Arabidopsis*. For these genes, we also analyzed physicochemical properties, phylogenetic and collinearity relationships, chromosomal localization, motif and gene structure compositions, and duplication analysis. Additionally, the gene ontology (GO) and Kyoto Encyclopedia of Genes and Genomics (KEGG) enrichment, cis-regulatory elements, and expression dynamics among various organs of grapevine have revealed extensive information related to the gene functions and their role in plant development. Subcellular predictions for maximum members of genes were largely found in diverse organelles, such as the nucleus, endoplasmic reticulum, cytoplasm, plasma membrane, mitochondria, and others. The physicochemical and protein properties (i.e., protein length (aa), molecular weight (kDa), isoelectric point (PIs), and grand average of hydropathicity (GRAVY)) drastically varied among PGs and PMEs, suggesting their variable role in micro and macro environments [33]. 

The selection pressure analysis (i.e., purifying, positive, and neutral selection) of gene pairs provides valuable information using the rate of divergence [34]. During evolutionary events, the values of *Ka/Ks* ratio that are less than 1.00 signify purifying selection; values equal to 1.00 specify neutral selection; and values greater than 1.00 shows positive selection [35,36]. In this study, we observed a strong selective pressure (purifying selection) among PGs compared to PMEs, which is in disagreement with the previously reported study on *Brassica rapa* [25]. We infer that PGs might duplicate earlier for their need and survival, intimating their diverse and variable functions. 

In this study, we also intended *Ka/Ks* values among 15 pairs of both PGs and PMEs (i.e., tandem, dispersed, and segmental) using the MEGA7.0 software [37]. Most of the pairs had *Ka/Ks* ratios that were smaller than 1.00, inferring the purifying selection. Furthermore, only four and five pairs of PGs and PMEs have values greater than 1.00, signifying positive selection. During evolutionary processes, the higher plant underwent polyploidization events, while segmental duplications are usually responsible for larger functional divergence [38,39]. Hence, studying gene duplication is vital for understanding biological functions and expansion of gene families [30,40], Hence, the results from our study highlight the importance of segmental and dispersed duplications in the two large families of PGs and PMEs in grapevines. 

Transcript expression patterns and abundance in particular organs at a given time provide clues for understanding the function of genes [28]. Transcriptional profiling and functional characterization of PGs and PMEs has been reported in several species. For example, the *PG* genes found in various species, including *Glycine max*, *Medicago truncatula*, *Zea mays,* and *O. sativa*, showed higher expression levels [41]. In strawberries, the downregulation of *FaPG1* extended their post-harvest life by reducing fruit softening [15]. Previously, PG gene family transcription patterns were also observed in *Arabidopsis* and *Populus* [24,42]. In addition, the members of the *PME* gene family have been reported as key regulators in plant–microbe interactions, cold acclimation, drought, and salt stress sensitivity [43,44,45,46,47]. Likewise, in grapevines, the members of the *PG* and *PME* gene family regulate the development of numerous organs at varying stages. In this study, 19 diverse tissue-specific expression patterns of *PG* and *PME* genes were examined. The results revealed that most PGs and PMEs were highly expressed in berry, tendril, and inflorescence. However, few PGs and PMEs reveal either more or similar expression patterns, signifying their unifying need and importance in plant development. In addition, qRT-PCR validation of 16 *PG* and *PME* genes revealed their vital role in various organs of grapevines (i.e., root, stem, tendril, inflorescence, flesh, skins, and leaves). Transcriptional profiling of these genes in various tissues may consequently aid the study of new adaptive functions regarding plant developmental processes in grapevine. Notably, among various *PG* and *PME* genes, visible tissue-specific expression patterns were detected, which may be correlated with their expansion, evolution and the complex nature in plant growth and development. However, their underlying molecular and evolutionary mechanisms that lead to the measurable and abrupt structural differences must be extensively investigated in the future. 

Finally, GO and KEGG enrichment, and cis-element predictions in the promoter regions of PGs and PMEs revealed their key role in pectin and carbohydrate metabolism, and various stress-related activities in grapevine. Taken together, our results highlight the importance of PGs and PMEs in plants and provides a comprehensive overview of their developmental role in grapevine. 

## 4. Materials and Methods 

### 4.1. Mining of Grapevine PGs and PMEs

For identification of *PG* and *PME* genes in grapevine (genome version 2.1), we used BioEdit tools to obtain PGs and PMEs from all the reference sequences of *Arabidopsis*. Grapevine and *Arabidopsis* genomic sequences were retrieved from Ensembl (https://plants.ensembl.org/index.html) and TAIR (http://www.arabidopsis.org/). The sequences of other species, including apple, peach, and citrus, were downloaded from Phytozome v12.1.6 (https://phytozome.jgi.doe.gov/pz/portal.html) [48]. For domain composition analysis, we used NCBI-Conserved Domain database (https://www.ncbi.nlm.nih.gov/Structure/cdd/wrpsb.cgi) and SMART databases (http://smart.embl-heidelberg.de/) [49]. In cases where PGs and PMEs domains were absent, the protein sequences were removed from the study and sequences with errors in length or having <100 aa length were also removed before analysis.

### 4.2. Phylogenetic Analysis of PGs and PMEs

The amino acid sequences of PGs and PMEs were aligned using MUSCLE [50] implemented in MEGA 7.0 software [51]. The phylogenetic trees were constructed using the maximum likelihood (ML) method in MEGA 7.0. In order to determine the reliability of the resulting trees, bootstrap values of 1000 replications were performed with the Jones, Taylor, and Thornton amino acid substitution model (JTT model).

### 4.3. Ratio of Synonymous (Ks) and Non-synonymous (Ka) for duplicated genes

The *Ka/Ks* ratios were calculated for duplicated pairs (i.e., tandem, dispersed, and segmental) using MEGA 7.0 [51]. The *Ka* and *Ks* substitution rates were calculated with the standard genetic code table by the Nei–Gojobori method (Jukes-Cantor model) in MEGA 7.0. 

### 4.4. Gene Structure, Conserved Motifs Analysis, and Physicochemical Parameters of PG and PME Proteins 

The gene structure was illustrated by TBtools software [52] by utilizing the GFF3 file of the grapevine genome. The conserved motif scanning of PG and PME proteins was carried out through local MEME Suite (Version 5.0.5) and was visualized by TBtools software. For this purpose, parameter settings were calibrated as follows: a maximum number of motifs of 10, with a minimum and maximum width of 50 and 100. The other parameters were set at default values [53]. The physicochemical properties of the PG and PME proteins (i.e., molecular weight (MW), isoelectronic points (PIs), aliphatic index and GRAVY values for each gene) were calculated using the ExPASY PROTPARAM tools (http://web.expasy.org/protparam/). The subcellular localization was predicted using the WOLF PSORT (https://wolfpsort.hgc.jp/) website.

### 4.5. Gene Ontology (GO), Kyoto Encyclopedia of Genes and Genomics (KEGG) and Cis-Elements Predictions of PGs and PMEs

The GO enrichment was carried using an online panther server (http://pantherdb.org/) and TBtools software [52]. KEGG enrichment analysis was carried out by the online server (https://www.genome.jp/kegg/pathway.html) and their enriched pathways were further analyzed by TBtools software [52]. The promoter sequences of PGs and PMEs (i.e., selected as 1500 bp) were imported in Generic File Format (GFF) file from the grapevine genome. Subsequently, the PlantCARE database (http://bioinformatics.psb.ugent.be/webtools/plantcare/html/) [54] was utilized for identifying various cis-regulatory elements for each promoter sequence of PGs and PMEs. 

### 4.6. Chromosomal Location and Collinearity Analysis

The chromosomal locations of PGs and PMEs were mapped based on information available at the Grape Genome Database (CRIBI. Available online: http://genomes.cribi.unipd.it/grape/, V2.1). and were illustrated using TBtools software [52]. For collinearity analysis, the relationships between grapevine and *Arabidopsis* homologs were verified and visualized by the Circos tool in TBtools software. 

### 4.7. Principal Component Analysis (PCA)

The principal component analysis was implemented using Rstudio (R program) for qRT-PCR at a significance level of 0.05 (*p*-value) [37,55].

### 4.8. Plant Material and Methods

Six-year-old *V. vinifera* cv. Summer Black plants grown under standard field conditions were selected from Jiangsu Academy of Agricultural Sciences (JAAS), Nanjing-China. In brief, the 4th unfolded leaf was selected for tissue extraction. Various grapevine tissues/organs, such as the root, stem, tendril, inflorescence, flesh, and skin, were collected at different developmental stages. Tissue samples were immediately frozen in liquid nitrogen and stored at −80 °C for further use and RNA extraction. 

### 4.9. RNA Isolation and Expression Profiling of PGs and PMEs in Grapevine

Total RNA was extracted from various organs using Trizol (Invitrogen, Carlsbad, CA, USA), following the manufacturer’s instructions. RNA was reverse-transcribed into cDNA using the Primer Script RT reagent kit (TAKARA, Dalian, China) according to the manufacturer’s instructions. Specific primers were designed using Becan Designer 7.9, and are presented in Table S7. In order to check the specificity of the primers, the BLAST tool was used against the grapevine genome for confirmation. RT-PCR was performed according to the guidelines of previous studies [56,57]. The relative fold expression was calculated with the comparative Ct-method. The expression patterns of all *PG* and *PME* genes were analyzed based on a previous study [58,59]. The housekeeping and grapevine actin gene (AB073011 and XM_010659103) was used as the reference gene for qRT-PCR. 

In brief, the RT-PCR amplification reactions were performed on an ABI 7500 RT-PCR System (Applied Biosystems, CA, USA) using SYBR Green (Applied Biosystems, Carlsbad, CA, USA) with three replicates. PCR was conducted as follows: denaturation at 95 °C for 2 min, 40 cycles of denaturation at 95 °C for 10 s, annealing at 60 °C for 40 s, and extension at 72 °C for 15 s, followed by melting curve analysis (61 cycles at 65 °C for 10 s).

Transcriptomic data were utilized for various organs and developmental stages from NCBI GEO server (https://www.ncbi.nlm.nih.gov/geo/) under the series entry GSE36128. Additionally, gene expression levels were quantified by FPKM (fragments per kilobase of transcript per million fragments mapped), and heat maps were visualized by using Rstudio (R program, Boston, MA, USA).

## 5. Conclusions

In conclusion, we systematically carried out a genome-wide exploration of grapevines through various bioinformatic analyses, which include elucidating the physicochemical properties of PGs and PMEs, phylogenetic characterization, collinearity of PGs and PMEs, gene structure and motif composition, evolutionary rates, and gene duplications. The GO and KEGG enrichment, and cis-elements prediction analysis extended our repositories on the putative functions of PGs and PMEs in plant developments during pectin and carbohydrate metabolism, and various stress-related activities in grapevine. Additionally, expression profiling of various organs during developmental stages and their correlation by principal component analysis highlights the essential role of PGs and PMEs for plant improvements in grapevines.

## Figures and Tables

**Table 1:** Duplications of the *PG* and *PME* genes in grapevine.

Gene 1	Gene 2	*Ks*	*Ka*	*Ka/Ks*	Selection Pressure	Gene Duplications
Between *PG* genes						
*VvPG1*	*VvPG2*	0.029	0.022	0.77	Purifying Selection	Tandem
*VvPG3*	*VvPG4*	0.034	0.016	0.47	Purifying Selection	Tandem
*VvPG6*	*VvPG25*	0.852	0.579	0.68	Purifying Selection	Tandem
*VvPG28*	*VvPG31*	0.738	0.693	0.94	Purifying Selection	Tandem
*VvPG33*	*VvPG34*	0.663	0.197	0.30	Purifying Selection	Tandem
*VvPG8*	*VvPG9*	0.5	0.458	0.92	Purifying Selection	Dispersed
*VvPG12*	*VvPG13*	0.626	0.58	0.93	Purifying Selection	Dispersed
*VvPG16*	*VvPG17*	0.879	0.472	0.54	Purifying Selection	Dispersed
*VvPG20*	*VvPG24*	1.56	0.419	0.27	Purifying Selection	Dispersed
*VvPG29*	*VvPG30*	0.522	0.628	1.20	Positive Selection	Dispersed
*VvPG5*	*VvPG7*	0.376	0.409	1.09	Positive Selection	WGD or Segmental
*VvPG10*	*VvPG11*	1.266	0.231	0.18	Purifying Selection	WGD or Segmental
*VvPG14*	*VvPG15*	0.212	0.343	1.62	Positive Selection	WGD or Segmental
*VvPG18*	*VvPG21*	0.515	0.522	1.01	Positive Selection	WGD or Segmental
*VvPG22*	*VvPG23*	1.401	0.152	0.11	Purifying Selection	WGD or Segmental
Between *PME* genes						
*VvPME2*	*VvPME3*	1.312	0.421	0.32	Purifying Selection	Dispersed
*VvPME5*	*VvPME16*	0.527	0.625	1.19	Positive Selection	Dispersed
*VvPME18*	*VvPME19*	1.102	0.442	0.40	Purifying Selection	Dispersed
*VvPME20*	*VvPME21*	0.881	0.464	0.53	Purifying Selection	Dispersed
*VvPME22*	*VvPME23*	0.446	0.681	1.53	Positive Selection	Dispersed
*VvPME27*	*VvPME28*	1.042	0.334	0.32	Purifying Selection	Dispersed
*VvPME35*	*VvPME36*	0.565	0.697	1.23	Positive Selection	Dispersed
*VvPME1*	*VvPME4*	0.798	0.438	0.55	Purifying Selection	Tandem
*VvPME8*	*VvPME10*	0.18	0.019	0.11	Purifying Selection	Tandem
*VvPME11*	*VvPME12*	0.879	0.086	0.10	Purifying Selection	Tandem
*VvPME13*	*VvPME15*	1.035	0.171	0.17	Purifying Selection	Tandem
*VvPME17*	*VvPME25*	0.64	0.631	0.99	Purifying Selection	Tandem
*VvPME6*	*VvPME7*	0.271	0.41	1.51	Positive Selection	WGD or Segmental
*VvPME14*	*VvPME26*	0.304	0.44	1.45	Positive Selection	WGD or Segmental
*VvPME33*	*VvPME34*	1.284	0.209	0.16	Purifying Selection	WGD or Segmental

## Supplementary Materials

The following are available online at https://www.mdpi.com/1422-0067/20/13/3180/s1.

## Abbreviations

PGs	Polygalacturonase
PMEs	Pectin methylesterase
GO	Gene Ontology
KEGG	Kyoto Encyclopedia of Genes and Genomes
RT-PCR	Real time-PCR
PCA	Principal Component Analysis
